# Interleukin-24 Induces Inflammatory Chemokine and Cytokine Responses in Fibroblast-Enriched Synovial Cells from Patients with Knee Osteoarthritis

**DOI:** 10.3390/biomedicines14092084

**Published:** 2026-09-16

**Authors:** Yui Uekusa, Kentaro Uchida, Makoto Itakura, Naoya Shibata, Manabu Mukai, Dai Iwase, Jun Aikawa, Ayumi Tsukada, Yukie Metoki, Gen Inoue, Masashi Takaso

**Affiliations:** 1 Department of Orthopaedic Surgery, School of Medicine, Kitasato University, 1-15-1 Minami-ku, Kitasato, Kanagawa, Sagamihara 252-0374, Japan; uekusa.yui@st.kitasato-u.ac.jp (Y.U.); shibata.naoya@st.kitasato-u.ac.jp (N.S.); mukai.manabu@kitasato-u.ac.jp (M.M.); daiiwase19760601@yahoo.co.jp (D.I.); jun43814@gmail.com (J.A.); amidesutarere9010@yahoo.co.jp (A.T.); yukiemetoki0826@gmail.com (Y.M.); ginoue@kitasato-u.ac.jp (G.I.); mtakaso@kitasato-u.ac.jp (M.T.); 2 Research Institute, Shonan University of Medical Sciences, Nishikubo 500, Kanagawa, Chigasaki 253-0083, Japan; 3 Department of Biochemistry, School of Medicine, Kitasato University, 1-15-1 Minami-ku, Kitasato, Sagamihara 252-0374, Japan; mitakura@med.kitasato-u.ac.jp

**Keywords:** knee osteoarthritis, synovium, fibroblast, synovial cell, interleukin-24

## Abstract

**Background:** Interleukin-24 (IL-24) is expressed in synovial myofibroblasts and has been associated with pain severity in female patients with knee osteoarthritis (OA). However, the direct effects of IL-24 on inflammatory responses in synovial fibroblasts and the signaling pathways involved remain unclear. This study investigated IL-24-induced transcriptional and inflammatory responses in fibroblast-enriched synovial cells and examined the effects of STAT3 and p38 mitogen-activated protein kinase (MAPK) inhibition. **Methods:** Fibroblast-enriched synovial cells isolated from synovial tissue obtained from patients with knee OA undergoing total knee arthroplasty were stimulated with IL-24 for 6 and 24 h. RNA sequencing (RNA-seq) was performed using cells from two patients, and overlapping upregulated genes were subjected to Kyoto Encyclopedia of Genes and Genomes pathway enrichment analysis. The concentration- and time-course experiment used samples from nine patients; the HJC0152 and SB203580 experiments used ten specimens from eight patients and ten specimens from nine patients, respectively. Selected inflammatory cytokines and chemokines were subsequently evaluated by quantitative polymerase chain reaction and enzyme-linked immunosorbent assay in concentration- and time-course experiments using nine specimens from nine patients. To investigate signaling involvement, cells were treated with IL-24 in the presence or absence of the STAT3 inhibitor HJC0152 or the p38 MAPK inhibitor SB203580, each at 5 or 10 μM. **Results:** RNA-seq identified 27 and 34 overlapping upregulated genes that independently met the DEG criteria in both patients at 6 and 24 h, respectively. These genes were enriched in inflammatory pathways, including TNF, IL-17, NF-κB, chemokine, cytokine–cytokine receptor interaction, Toll-like receptor, and NOD-like receptor signaling pathways. IL-24 significantly increased the mRNA expression of *CCL2*, *CXCL1*, *CXCL3*, *IL6*, and *IL8*. HJC0152 and SB203580 at 10 μM both significantly reduced IL-24-associated *CCL2*, *CXCL1*, and *IL6* expression. At the protein level, both inhibitors attenuated CCL2, CXCL1, and IL-6 concentrations in IL-24-stimulated cells. HJC0152 also reduced *CXCL3* expression at the mRNA level, although its protein-level effect was less pronounced. In contrast, the effects of both inhibitors on CXCL3, CXCL10, and IL-8 were limited or inconsistent. **Conclusions:** IL-24 induces an inflammatory response in fibroblast-enriched synovial cells characterized by increased chemokine and cytokine expression. STAT3 and p38 MAPK inhibition preferentially attenuated IL-24-associated CCL2, CXCL1, and IL-6 responses, suggesting the possible involvement of these pathways in selected IL-24-associated inflammatory responses. These findings provide a basis for further investigation of IL-24-related signaling in OA synovitis.

## 1. Introduction

Osteoarthritis (OA) is a whole-joint disease involving articular cartilage, subchondral bone, synovium, and periarticular tissues [1]. Although cartilage degeneration is a central feature of OA, synovitis is increasingly recognized as an important component of disease pathophysiology and is associated with clinical symptoms and structural progression [2,3,4]. Within the synovium, fibroblasts represent a major stromal cell population and can contribute to the local inflammatory environment through the production of cytokines, chemokines, and matrix-remodeling mediators [5,6].

Interleukin-24 (IL-24) is a member of the IL-20 cytokine subfamily within the IL-10 family and has context-dependent immunoregulatory functions [7]. Its relevance to joint inflammation has been suggested in rheumatoid arthritis (RA) and spondyloarthritis (SpA). Kragstrup et al. reported increased IL-24 concentrations in synovial fluid and plasma from patients with RA and SpA, together with positive associations between IL-24 concentrations and CCL2/MCP-1 concentrations [8]. In the same study, recombinant IL-24 increased CCL2/MCP-1 secretion from synovial fluid mononuclear cells [8]. These findings suggest that IL-24 can promote inflammatory chemokine responses within the joint; however, its direct effects on synovial fibroblasts derived from patients with OA remain poorly understood.

In our previous study, *IL24* expression was elevated in synovial tissue from female patients with knee OA, positively correlated with pain severity in women, and enriched in a myofibroblast-rich synovial fraction [9]. These findings identified IL-24 as a synovial factor associated with a distinct OA synovial phenotype. However, whether IL-24 directly induces inflammatory responses in OA-derived synovial fibroblasts and which signaling pathways contribute to these responses remain unclear.

IL-24 signals through the heterodimeric IL-20R1/IL-20R2 and IL-22R1/IL-20R2 receptor complexes [10,11]. Ligands binding to these receptor complexes can activate JAK/STAT signaling, including STAT3 [11,12]. In human pancreatic myofibroblasts expressing all three receptor subunits, recombinant IL-24 rapidly induced p38 MAPK as well as STAT1/3 phosphorylation [13]. IL-24 also increased p38 phosphorylation in human monocyte-derived dendritic cells [14]. However, the involvement of STAT3 and p38 MAPK in IL-24-associated inflammatory responses in OA synovial fibroblasts has not been defined.

Therefore, this study aimed to characterize IL-24-induced inflammatory responses in fibroblast-enriched synovial cells derived from patients with knee OA and to examine the potential involvement of STAT3 and p38 MAPK signaling. Specifically, we sought to identify inflammatory mediators induced by IL-24 and to determine whether their expression was attenuated by pharmacological inhibition of STAT3 or p38 MAPK.

## 2. Materials and Methods

### 2.1. Patients and Synovial Tissue Collection

Synovial tissues were obtained from 22 knees of 20 patients with symptomatic radiographic knee OA (3 men and 17 women; age range, 56–85 years; BMI range, 19.7–33.8 kg/m^2^) who underwent total knee arthroplasty at Kitasato University Hospital. OA was diagnosed by orthopedic surgeons on the basis of knee pain, physical examination, and radiographic findings. Radiographic severity was classified using the Kellgren–Lawrence (KL) grading system; all included knees were KL grades 2–4 and therefore showed definite osteophyte formation, with definite joint-space narrowing present in KL grades 3–4. Patients with rheumatoid arthritis, autoimmune disease, inflammatory arthropathy, or other systemic joint disorders were excluded.

Samples from nine patients (seven women and two men) were used for the IL-24 concentration- and time-course experiment. Cells from two patients (one woman and one man) were used for RNA-seq, one of whom was also included in the concentration- and time-course experiment. The HJC0152 experiment included ten knee synovial specimens from eight women, whereas the SB203580 experiment included ten knee synovial specimens from nine women. Two women in the HJC0152 cohort and one woman in the SB203580 cohort contributed bilateral-knee specimens. Patient characteristics and allocation of synovial specimens to each experiment are provided in Table S1.

The study protocol was approved by the Institutional Review Board of Kitasato University (Approval No. B19-259; approved on 27 January 2020) and was conducted in accordance with the Declaration of Helsinki. An opt-out consent procedure was used, as approved by the Institutional Review Board.

### 2.2. Isolation of a Synovial Fibroblast-Enriched Cell Population

A synovial fibroblast-enriched cell population was isolated from freshly obtained synovial tissues using a magnetic bead-based negative selection method. Synovial tissues were minced into small fragments and digested overnight at 37 °C with 400 U/mL collagenase type I (FUJIFILM Wako Pure Chemical Corporation, Osaka, Japan) in α-minimum essential medium (α-MEM; Invitrogen, Carlsbad, CA, USA) supplemented with 1% fetal bovine serum (FBS). The digested tissue was passed through a 100 μm cell strainer, and the resulting cell suspension was centrifuged and resuspended in α-MEM supplemented with 1% FBS.

The cells were incubated for 30 min at 4 °C with biotin-conjugated anti-CD45 and anti-CD31 antibodies (BioLegend, San Diego, CA, USA) to deplete leukocytes and endothelial cells, respectively. After washing with α-MEM supplemented with 1% FBS, the cells were incubated with streptavidin-coated magnetic particles (BD Biosciences, Milpitas, CA, USA) for 30 min at 4 °C. The cell suspension was then placed in an IMag magnetic separation system (BD Biosciences) for 8 min at room temperature. The negative fraction, depleted of CD45-positive leukocytes and CD31-positive endothelial cells, was collected as fibroblast-enriched synovial cells and used for subsequent experiments. In a previous study using the same isolation procedure, flow cytometry demonstrated that >90% of the isolated cells were CD45−CD90+ [15]. Positive-marker analysis was not repeated for the specimens used in the present study.

### 2.3. Cell Culture and Stimulation

Fibroblast-enriched synovial cells were cultured in 75 cm^2^ tissue culture flasks in α-MEM supplemented with 10% FBS, 1% penicillin–streptomycin, and 20 ng/mL basic fibroblast growth factor (bFGF; Kaken Pharmaceutical Co., Ltd., Tokyo, Japan) at 37 °C in a humidified atmosphere containing 5% CO_2_. When the passage-0 (P0) cells reached approximately 80% confluence, they were detached using 0.25% trypsin/ethylenediaminetetraacetic acid (EDTA; Invitrogen) and seeded into 6-well plates at a density of 2 × 10^5^ cells per well as passage-1 (P1) cells. Three days after seeding, the cells were used for subsequent experiments.

Because *CCL2* has been reported to be induced by IL-24 [16], it was selected as a readout for establishing rhIL-24 stimulation conditions. Fibroblast-enriched synovial cells were treated with rhIL-24 (R&D Systems, Minneapolis, MN, USA) at concentrations of 0, 10, or 100 ng/mL for 1, 3, 6, and 24 h. Total RNA was collected at each time point for reverse transcription-quantitative polymerase chain reaction (RT-qPCR) analysis of *CCL2* mRNA expression. Culture supernatants collected after 24 h of treatment were used to measure CCL2 protein concentrations by enzyme-linked immunosorbent assay (ELISA). Based on the mRNA and protein responses of CCL2, 100 ng/mL rhIL-24 was selected for all subsequent experiments (Figure S1). For RNA-seq analysis, cells were treated with 100 ng/mL rhIL-24 for 6 and 24 h.

Based on the established involvement of STAT3 in IL-24 receptor signaling and reported links between p38 MAPK and IL-24-related cellular responses [13,14], we examined the effects of pharmacological inhibition of these pathways on IL-24-induced inflammatory responses in fibroblast-enriched synovial cells. For inhibitor experiments, cells were pretreated for 30 min with the STAT3 inhibitor HJC0152 or the p38 MAPK inhibitor SB203580, each at a final concentration of 5 or 10 μM. HJC0152 and SB203580 were purchased from Selleck Chemicals (Houston, TX, USA). After pretreatment, the cells were stimulated with 100 ng/mL rhIL-24 for 24 h. Vehicle-treated cells cultured in α-MEM containing 1% FBS served as controls. After treatment, total RNA and culture supernatants were collected for RT-qPCR and ELISA, respectively, to evaluate the inflammatory cytokines and chemokines identified as upregulated by RNA-seq.

### 2.4. Reverse Transcription-Quantitative Polymerase Chain Reaction

Total RNA was extracted using TRIzol reagent (Invitrogen, Carlsbad, CA, USA) and purified using the Direct-zol RNA MicroPrep Kit (Cat. No. R2062; Zymo Research, Irvine, CA, USA) according to the manufacturer’s instructions. RNA concentration was determined using a spectrophotometer (DeNovix, Wilmington, DE, USA). First-strand complementary DNA (cDNA) was synthesized from purified total RNA using the SuperScript III First-Strand Synthesis System (Invitrogen).

Quantitative PCR was performed using TB Green Premix Ex Taq II (Takara Bio Inc., Shiga, Japan) on a CFX Connect Real-Time PCR Detection System (Bio-Rad Laboratories, Hercules, CA, USA). Primers were designed using Primer-BLAST and purchased from Hokkaido System Science Co., Ltd. (Sapporo, Japan). Primer sequences used in this study are listed in Table 1. *GAPDH* was used as the endogenous control. Relative gene expression was calculated using the 2^−^ΔΔCt method, with the corresponding vehicle-treated control group set to 1.0.

### 2.5. RNA Sequencing and Bioinformatic Analysis

Fibroblast-enriched synovial cells obtained from two patients were treated with vehicle or 100 ng/mL rhIL-24 for 6 or 24 h. Total RNA was extracted as described above. RNA concentration was determined using a spectrophotometer (DeNovix, Wilmington, DE, USA), and RNA quality was assessed using an Agilent 2100 Bioanalyzer with an RNA 6000 Nano Chip (Agilent Technologies, Santa Clara, CA, USA).

RNA-seq was performed using an MGI DNBSEQ-G400 sequencer (BGI, Shenzhen, China). Because only two patients were included, between-patient variability could not be reliably estimated for a conventional group-level differential-expression analysis. Treated-versus-vehicle read counts were therefore compared separately within each patient and at each time point using the PoissonDis method implemented in the Dr. Tom Multi-omics Data Mining System (BGI) [17,18]. *p* values were adjusted for multiple testing to control the false-discovery rate. Patient-specific candidate DEGs were defined by |log_2_ fold change| ≥ 1 and an adjusted *p* value < 0.05. Genes satisfying these criteria independently in both patients at the same time point were designated overlapping candidate DEGs. KEGG pathway enrichment analysis was performed only on the overlapping upregulated gene sets.

### 2.6. Enzyme-Linked Immunosorbent Assay

Culture supernatants were collected after 24 h of treatment and stored at −30 °C until analysis. The concentrations of CCL2, CXCL1, CXCL3, CXCL10, IL-6, and IL-8 in culture supernatants were measured using commercially available ELISA kits according to the manufacturers’ instructions. The CXCL1 ELISA kit was purchased from Sigma-Aldrich (St. Louis, MO, USA), and the CXCL3 ELISA kit was purchased from Abcam (Cambridge, UK). ELISA kits for CCL2, CXCL10, IL-6, and IL-8 were purchased from BioLegend (San Diego, CA, USA).

### 2.7. Statistical Analysis

Normality was assessed using the Shapiro–Wilk test. Because the data were not consistently normally distributed, nonparametric statistical tests were used. Data are presented as box-and-whisker plots. Because cells derived from each synovial specimen were evaluated under all treatment conditions, measurements were matched within specimens. Differences among multiple treatment conditions were therefore analyzed using the Friedman test followed by Dunn’s multiple-comparisons test. A two-sided *p* value < 0.05 was considered statistically significant. Statistical analyses were performed using IBM SPSS Statistics for Windows, version 28.0 (IBM Corp., Armonk, NY, USA).

## 3. Results

### 3.1. IL-24 Induces an Inflammatory Transcriptional Response in Fibroblast-Enriched Synovial Cells

**Figure 1:**
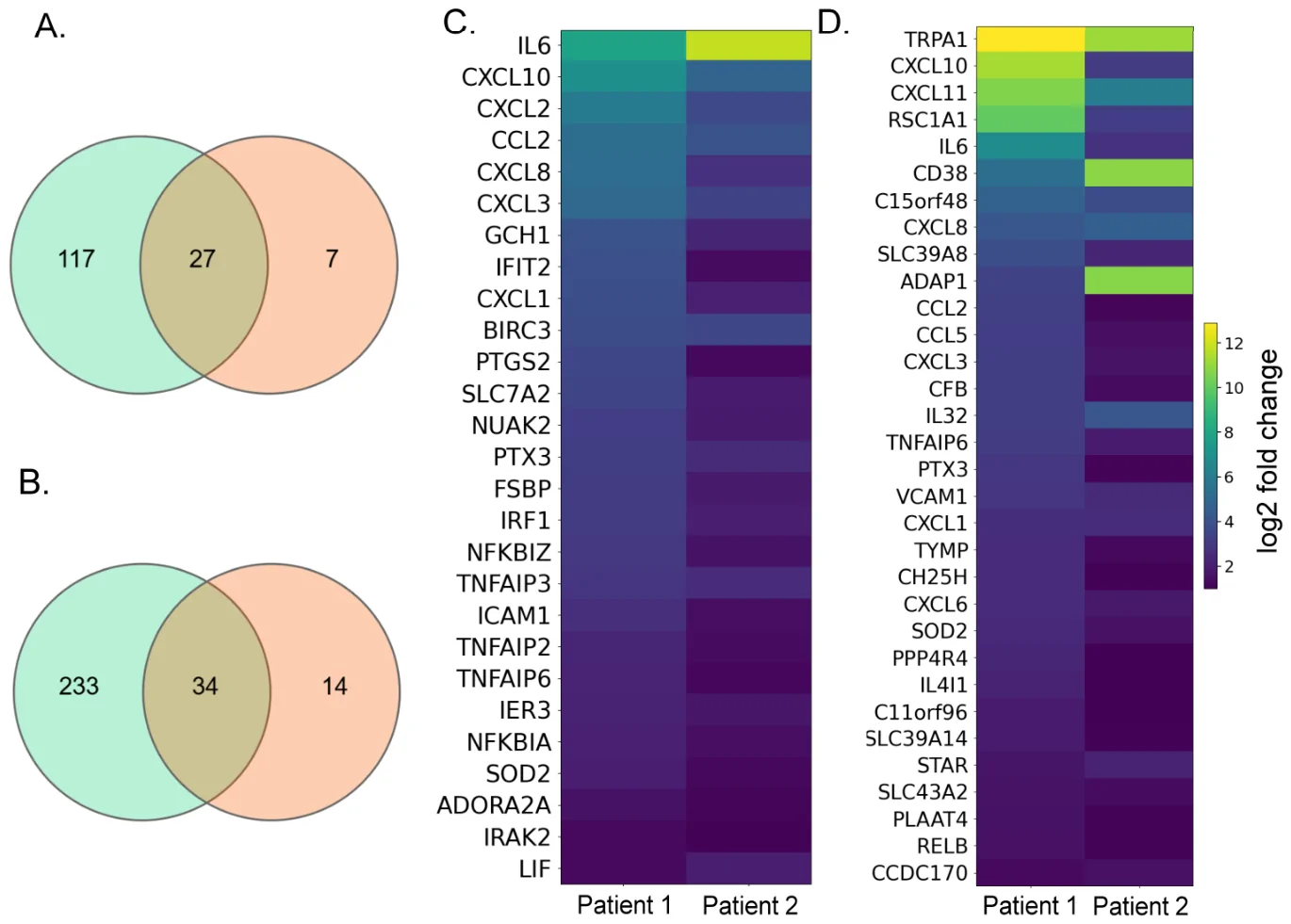
Overlapping upregulated genes identified by RNA-seq after IL-24 stimulation. (**A**,**B**) Venn diagrams showing the overlap of upregulated candidate DEGs identified independently in the two patients by comparing IL-24-treated cells with vehicle-treated cells after 6 h (**A**) and 24 h (**B**). (**C**,**D**) Heat maps showing the log_2_ fold changes of the overlapping upregulated genes in each patient at 6 h (**C**) and 24 h (**D**). Genes meeting the prespecified differential-expression criteria independently in both patients at the same time point were defined as overlapping candidate DEGs.

**Figure 2:**
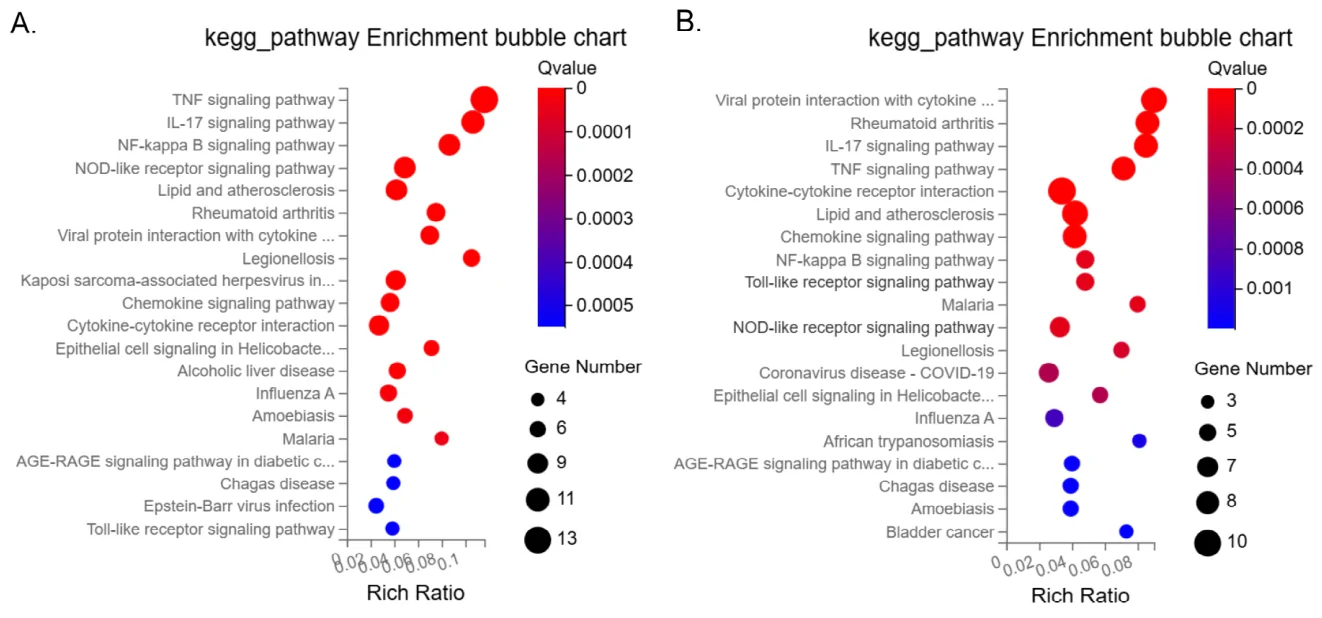
KEGG pathway enrichment analysis of overlapping upregulated genes after IL-24 stimulation. (**A**,**B**) Kyoto Encyclopedia of Genes and Genomes (KEGG) pathway enrichment analysis of the overlapping upregulated genes identified after 6 h (**A**) and 24 h (**B**) of IL-24 stimulation. Pathway labels truncated by the plotting software correspond to the following full names: epithelial cell signaling in Helicobacter pylori infection; AGE-RAGE signaling pathway in diabetic complications; viral protein interaction with cytokine and cytokine receptor; and Kaposi sarcoma-associated herpesvirus infection.

RNA-seq analysis was performed using samples from two patients after IL-24 stimulation for 6 and 24 h. At each time point, IL-24-treated and vehicle-treated samples were compared separately within each patient, and genes meeting the prespecified DEG criteria independently in both patients were defined as overlapping upregulated candidate DEGs. This analysis identified 27 overlapping upregulated candidate DEGs at 6 h and 34 at 24 h (Figure 1A–D, Tables S2 and S3). KEGG pathway enrichment analysis of the overlapping upregulated genes demonstrated significant enrichment of inflammatory and immune-related pathways. At 6 h, enriched pathways included the TNF signaling pathway, IL-17 signaling pathway, NF-κB signaling pathway, NOD-like receptor signaling pathway, chemokine signaling pathway, cytokine–cytokine receptor interaction, and rheumatoid arthritis pathway (Figure 2A). At 24 h, the enriched pathways included IL-17 signaling, TNF signaling, cytokine–cytokine receptor interaction, chemokine signaling, NF-κB signaling, Toll-like receptor signaling, and NOD-like receptor signaling pathways (Figure 2B).

These exploratory findings indicated concordant inflammatory transcriptional responses to IL-24 in both patients. For subsequent validation by qPCR and ELISA, we selected *IL6*, CXCL8 (*IL8)*, *CCL2*, *CXCL1*, *CXCL3*, and *CXCL10* because these genes were upregulated in both patients at both time points and encode secreted inflammatory mediators.

Given the established involvement of STAT3 in IL-24 receptor signaling and evidence of IL-24-induced p38 MAPK phosphorylation in other cell types [13,14], we next examined the effects of pharmacological inhibition of these pathways using the STAT3 inhibitor HJC0152 and the p38 MAPK inhibitor SB203580. The mRNA expression and protein concentrations of these inflammatory mediators in culture supernatants were subsequently evaluated by qPCR and ELISA, respectively.

### 3.2. Effects of STAT3 Inhibition by HJC0152 on IL-24-Associated Chemokine and Inflammatory Cytokine Expression

**Figure 3:**
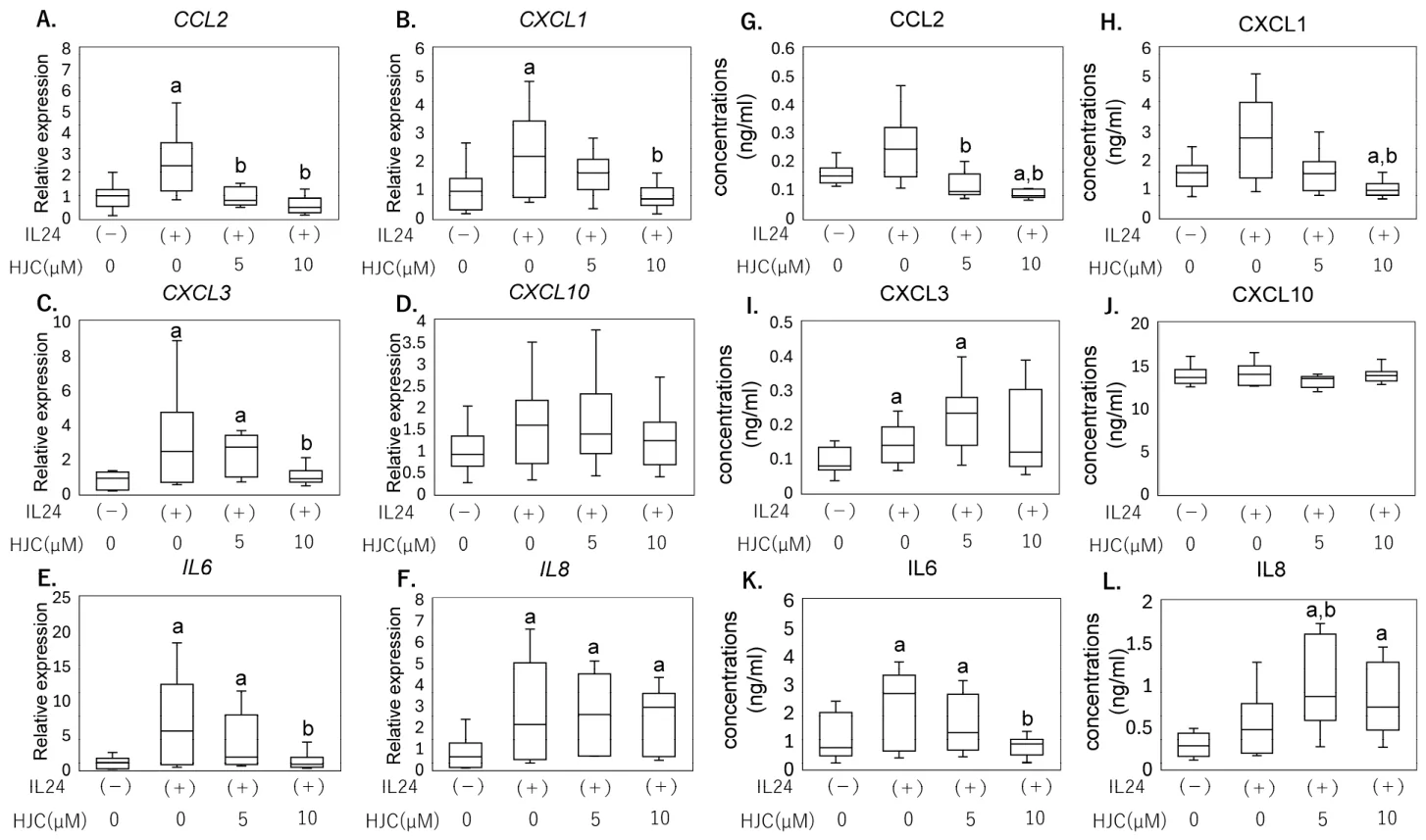
Effects of the STAT3 inhibitor HJC0152 on IL-24-induced inflammatory cytokine and chemokine expression in fibroblast-enriched synovial cells. Fibroblast-enriched synovial cells were treated with vehicle or 100 ng/mL recombinant human IL-24 (rhIL-24) in the presence or absence of HJC0152 (5 or 10 μM) for 24 h. (**A**–**F**) Relative mRNA expression of *CCL2*, *CXCL1*, *CXCL3*, *CXCL10*, *IL6*, and *IL8*, as assessed by RT-qPCR. (**G**–**L**) Concentrations of CCL2, CXCL1, CXCL3, CXCL10, IL-6, and IL-8 in culture supernatants, as measured by ELISA. Data are presented as box-and-whisker plots. Each experiment used synovial cells derived from 10 specimens from 8 patients with knee OA. ^a^ *p* < 0.05 vs. vehicle; ^b^ *p* < 0.05 vs. IL-24 alone.

Fibroblast-enriched synovial cells were cultured with medium alone (control), IL-24 alone, or IL-24 in combination with HJC0152 at 5 or 10 μM (Figure 3).

At the mRNA level, significant overall differences among the treatment groups were observed for *CCL2*, *CXCL1*, *CXCL3*, *IL6*, and *IL8* (all Friedman test, *p* < 0.001), whereas no significant difference was observed for *CXCL10* (*p* = 0.871). Compared with the control group, IL-24 significantly increased the expression of *CCL2* (*p* = 0.002), *CXCL1* (*p* < 0.001), *CXCL3* (*p* < 0.001), *IL6* (*p* < 0.001), and *IL8* (*p* = 0.013). Compared with IL-24 alone, HJC0152 at 10 μM significantly reduced the expression of *CCL2* (*p* < 0.001), *CXCL1* (*p* < 0.001), *CXCL3* (*p* = 0.040), and *IL6* (*p* < 0.001). The expression levels of *CCL2*, *CXCL1*, *CXCL3*, and *IL6* in the HJC0152 10 μM group did not differ significantly from those in the control group (*p* = 0.137, *p* = 0.603, *p* = 0.206, and *p* = 0.869, respectively). HJC0152 at 5 μM significantly reduced *CCL2* expression compared with IL-24 alone (*p* = 0.021), but did not significantly affect *CXCL1*, *CXCL3*, or *IL6* expression (*p* = 0.119, *p* = 0.752, and *p* = 0.620, respectively). *IL8* expression remained significantly higher than that in the control group in both the 5 μM (*p* < 0.001) and 10 μM (*p* = 0.001) HJC0152 groups. However, neither 5 nor 10 μM HJC0152 significantly altered *IL8* expression compared with IL-24 alone (*p* = 0.160 and *p* = 0.457, respectively).

At the protein level, significant overall differences among the treatment groups were observed for CCL2 (*p* < 0.001), CXCL1 (*p* < 0.001), CXCL3 (*p* = 0.006), IL-6 (*p* < 0.001), and IL-8 (*p* < 0.001), whereas no significant overall difference was observed for CXCL10 (*p* = 0.086). IL-24 alone did not significantly increase CCL2 or CXCL1 concentrations compared with the control group (*p* = 0.057 and *p* = 0.119, respectively). Nevertheless, both 5 and 10 μM HJC0152 significantly reduced CCL2 concentrations compared with IL-24 alone (*p* = 0.015 and *p* < 0.001, respectively). CCL2 concentrations in the 10 μM HJC0152 group were significantly lower than those in the control group (*p* = 0.006), whereas those in the 5 μM HJC0152 group did not differ significantly from control levels (*p* = 0.603). For CXCL1, HJC0152 at 10 μM significantly reduced protein concentrations compared with IL-24 alone (*p* < 0.001). For CXCL3, IL-24 significantly increased protein concentrations compared with the control group (*p* = 0.009). HJC0152 at 10 μM showed a trend toward lower CXCL3 concentrations compared with IL-24 alone (*p* = 0.057), whereas HJC0152 at 5 μM had no significant effect (*p* = 0.729). CXCL3 concentrations in the 10 μM HJC0152 group did not differ significantly from control levels (*p* = 0.488), whereas those in the 5 μM HJC0152 group remained significantly higher than control levels (*p* = 0.003). IL-24 significantly increased IL-6 concentrations compared with the control group (*p* = 0.002). HJC0152 at 10 μM significantly reduced IL-6 concentrations compared with IL-24 alone (*p* < 0.001), and IL-6 concentrations in this group did not differ significantly from control levels (*p* = 0.862). In contrast, HJC0152 at 5 μM did not significantly reduce IL-6 concentrations relative to IL-24 alone (*p* = 0.603), and IL-6 concentrations remained significantly higher than those in the control group (*p* = 0.009). For IL-8, IL-24 alone showed a trend toward increased concentrations compared with the control group (*p* = 0.057). HJC0152 at 5 μM significantly increased IL-8 concentrations compared with IL-24 alone (*p* = 0.002), whereas the 10 μM HJC0152 group did not differ significantly from the IL-24 group (*p* = 0.119). IL-8 concentrations in both HJC0152-treated groups were significantly higher than those in the control group (both *p* < 0.001).

Collectively, HJC0152 at 10 μM consistently attenuated IL-24-associated CCL2, CXCL1, and IL-6 responses at both the mRNA and protein levels. HJC0152 also reduced *CXCL3* expression at the mRNA level, whereas its effect on CXCL3 protein concentrations did not reach statistical significance. In contrast, HJC0152 did not suppress the IL-24-associated IL-8 response.

### 3.3. Effects of P38 MAPK Inhibition by SB203580 on IL-24-Associated Chemokine and Inflammatory Cytokine Expression

**Figure 4:**
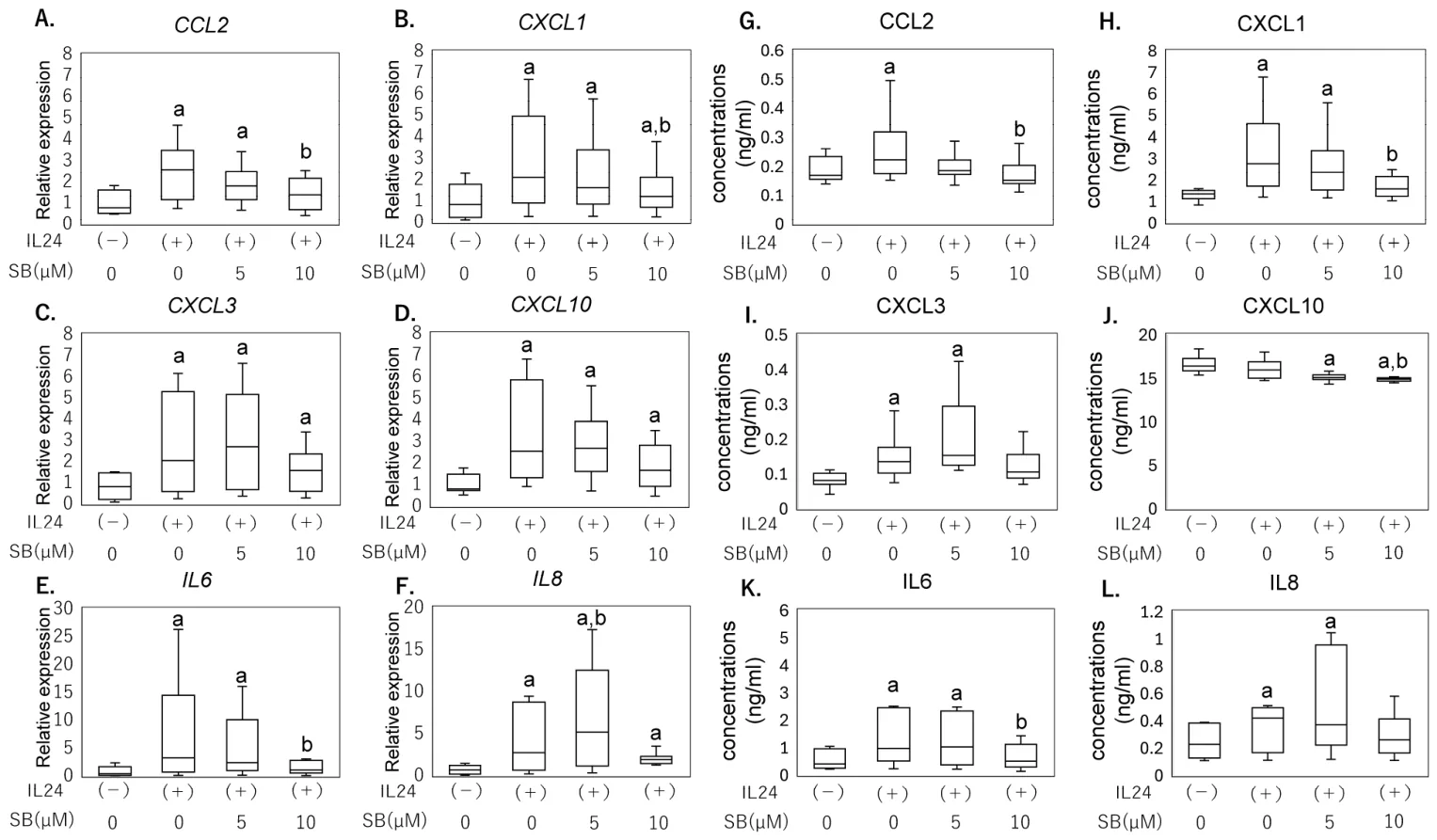
Effects of the p38 MAPK inhibitor SB203580 on IL-24-induced inflammatory cytokine and chemokine expression in fibroblast-enriched synovial cells. Fibroblast-enriched synovial cells were treated with vehicle or 100 ng/mL recombinant human IL-24 (rhIL-24) in the presence or absence of SB203580 (5 or 10 μM) for 24 h. (**A**–**F**) Relative mRNA expression of *CCL2*, *CXCL1*, *CXCL3*, *CXCL10*, *IL6*, and *IL8*, assessed by RT-qPCR. (**G**–**L**) Concentrations of CCL2, CXCL1, CXCL3, CXCL10, IL-6, and IL-8 in culture supernatants, measured by ELISA. Data are presented as box-and-whisker plots from synovial cells derived from 10 specimens from nine patients with knee OA. ^a^ *p* < 0.05 vs. vehicle; ^b^ *p* < 0.05 vs. IL-24 alone.

Fibroblast-enriched synovial cells were cultured with medium alone (control), IL-24 alone, or IL-24 in combination with SB203580 at 5 or 10 μM (Figure 4).

At the mRNA level, significant overall differences among the treatment groups were observed for *CCL2*, *CXCL1*, *CXCL3*, *CXCL10*, *IL6*, and *IL8* (all Friedman test *p* < 0.001). Compared with the control group, IL-24 significantly increased the expression of *CCL2*, *CXCL1*, *CXCL3*, *CXCL10*, and *IL6* (all *p* < 0.001), as well as *IL8* (*p* = 0.038) compared with IL-24 alone. SB203580 at 10 μM significantly reduced the expression of *CCL2* (*p* = 0.006), *CXCL1* (*p* = 0.024), and *IL6* (*p* = 0.006). *CCL2* and *IL6* expression levels in the 10 μM SB203580 group did not differ significantly from those in the control group (*p* = 0.083 and *p* = 0.166, respectively), whereas *CXCL1* expression remained significantly higher than control levels (*p* = 0.038). SB203580 at 5 μM did not significantly reduce IL-24-associated *CCL2*, *CXCL1*, or *IL6* expression (*p* = 0.299, *p* = 0.729, and *p* = 1.000, respectively).

Neither concentration of SB203580 significantly reduced *CXCL3* or *CXCL10* expression compared with IL-24 alone. For *CXCL3*, the corresponding *p* values were 0.488 and 0.166 for 5 and 10 μM SB203580, respectively. For *CXCL10*, the corresponding *p* values were 0.795 and 0.141. In the 10 μM SB203580 group, *CXCL3* and *CXCL10* expression remained significantly higher than control levels (*p* = 0.038 and *p* = 0.030, respectively). For *IL8*, SB203580 at 5 μM significantly increased expression compared with IL-24 alone (*p* = 0.003), whereas SB203580 at 10 μM did not significantly alter *IL8* expression (*p* = 0.225).

At the protein level, significant overall differences among the treatment groups were observed for CCL2 (*p* < 0.001), CXCL1 (*p* < 0.001), CXCL3 (*p* < 0.001), CXCL10 (*p* < 0.001), IL-6 (*p* < 0.001), and IL-8 (*p* = 0.005). Compared with the control group, IL-24 significantly increased the concentrations of CCL2 (*p* = 0.001), CXCL1 (*p* < 0.001), CXCL3 (*p* = 0.006), IL-6 (*p* = 0.003), and IL-8 (*p* = 0.038). In contrast, IL-24 alone did not significantly alter CXCL10 concentrations compared with the control group (*p* = 0.083). CXCL10 concentrations were significantly lower than control levels in both the 5 and 10 μM SB203580 groups (*p* < 0.001 and *p* < 0.001, respectively).

SB203580 at 10 μM significantly reduced CCL2, CXCL1, and IL-6 concentrations compared with IL-24 alone (all *p* < 0.001). CCL2, CXCL1, and IL-6 concentrations in the SB203580 10 μM group did not differ significantly from those in the control group (*p* = 0.341, *p* = 0.729, and *p* = 0.386, respectively).

Although SB203580 at 10 μM did not significantly reduce CXCL3 concentrations compared with IL-24 alone (*p* = 0.119), CXCL3 concentrations in this group did not differ significantly from control levels (*p* = 0.225). SB203580 at 10 μM significantly reduced CXCL10 concentrations compared with IL-24 alone (*p* = 0.038), and CXCL10 concentrations in the SB203580 10 μM group were also significantly lower than those in the control group (*p* < 0.001).

Neither 5 nor 10 μM SB203580 significantly altered IL-8 concentrations compared with IL-24 alone (*p* = 0.299 and *p* = 0.083, respectively). IL-8 concentrations in the 10 μM SB203580 group did not differ significantly from those in the control group (*p* = 0.729).

Collectively, SB203580 at 10 μM attenuated IL-24-associated CCL2, CXCL1, and IL-6 responses in fibroblast-enriched synovial cells. In contrast, its effects on CXCL3 and IL-8 were limited or inconsistent across mRNA and protein analyses. Although SB203580 reduced CXCL10 protein concentrations, IL-24 alone did not increase CXCL10 protein concentrations, making this effect difficult to attribute specifically to inhibition of an IL-24-associated response.

Together, these pharmacological findings suggest that STAT3- and p38 MAPK-related signaling may contribute to selected IL-24-associated responses, particularly those involving CCL2, CXCL1, and IL-6.

## 4. Discussion

In the present study, IL-24 induced an inflammatory transcriptional response in the fibroblast-enriched synovial cells derived from patients with knee OA. RNA-seq analysis demonstrated enrichment of pathways related to TNF signaling, IL-17 signaling, NF-κB signaling, chemokine signaling, cytokine–cytokine receptor interaction, Toll-like receptor signaling, and NOD-like receptor signaling. Targeted analyses further showed that IL-24 increased the expression of *CCL2*, *CXCL1*, *CXCL3*, *IL6*, and *IL8*. In addition, pharmacological inhibition of STAT3 with HJC0152 or p38 MAPK with SB203580 preferentially attenuated IL-24-associated CCL2, CXCL1, and IL-6 responses. These findings suggest that STAT3- and p38 MAPK-related signaling may contribute, at least in part, to IL-24-induced inflammatory responses. Collectively, our findings indicate that IL-24 can induce inflammatory mediator expression in OA-derived synovial fibroblasts.

Synovitis is increasingly recognized as an important component of OA and is associated with clinical symptoms and structural progression [3,4,19]. Synovial fibroblasts are major stromal cells within the synovium and can contribute to the local inflammatory environment through the production of cytokines and chemokines [5,6,20,21]. In this context, the present findings identify IL-24 as a cytokine capable of inducing inflammatory responses in synovial fibroblasts from patients with OA.

Our previous study showed that *IL24* expression was elevated in synovial tissue from female patients with knee OA, positively correlated with pain severity in female patients, and was enriched in a myofibroblast-rich synovial fraction [9]. The present study extends these observations by demonstrating that IL-24 directly induces inflammatory mediator expression in synovial fibroblasts. Although the present experiments do not establish the in vivo role of IL-24 in OA synovium, they provide functional evidence that synovial fibroblasts can respond to IL-24 with increased expression of inflammatory cytokines and chemokines.

The induction of CCL2 is consistent with previous studies of inflammatory arthritis. Kragstrup et al. reported increased IL-24 concentrations in synovial fluid and plasma from patients with rheumatoid arthritis and spondyloarthritis, together with positive associations between IL-24 and CCL2/MCP-1 concentrations [8]. Consistent with our findings, previous work showed that recombinant IL-24 enhanced CCL2/MCP-1 production in synovial fluid mononuclear cells [8]. A subsequent study in spondyloarthritis further showed that IL-24 increased CCL2 production in activated synovial fluid monocytes [16]. Our results extend these observations to OA-derived synovial fibroblasts. Notably, the previous inflammatory arthritis study did not detect IL-24-induced IL-6 production in synovial fluid mononuclear cell cultures [8], whereas IL-24 increased *IL6* expression and IL-6 secretion in the present fibroblast-enriched synovial cell cultures. This difference suggests that downstream responses to IL-24 may vary according to cellular context.

IL-24 also increased *CXCL1*, *CXCL3*, *IL6*, and *IL8* expression. Together with CCL2, these mediators are components of the inflammatory synovial milieu in OA [20,22,23,24]. The simultaneous induction of several chemokines and cytokines supports the RNA-seq findings indicating a broad inflammatory transcriptional response to IL-24. However, the present study did not evaluate leukocyte migration, fibroblast–immune cell interactions, cartilage catabolism, or pain-related responses. Therefore, the functional consequences of individual IL-24-induced mediators in OA require further investigation.

IL-24 belongs to the IL-20 cytokine subfamily and signals through the IL-20R1/IL-20R2 or IL-22R1/IL-20R2 receptor complexes [7,10,12]. These receptor complexes can activate JAK/STAT-associated signaling, including STAT3 [10,12,25]. In the present study, HJC0152 attenuated IL-24-associated CCL2, CXCL1, and IL-6 responses, which is consistent with a contribution of STAT3 signaling to selected IL-24-induced inflammatory responses. However, STAT3 activation and IL-24 receptor expression were not directly assessed. Therefore, the present pharmacological findings are consistent with the possible involvement of STAT3-related signaling but do not establish the precise receptor-to-STAT3 signaling sequence in OA-derived synovial fibroblasts.

The effects of p38 MAPK inhibition should also be interpreted cautiously. Recombinant IL-24 has been reported to induce p38 phosphorylation in other cell types [13,14]. In the present study, SB203580 attenuated IL-24-associated CCL2, CXCL1, and IL-6 responses. However, because p38 phosphorylation, *IL24* expression after inhibitor treatment, and temporal pathway activation were not examined, the present results do not determine whether p38 MAPK acts directly downstream of IL-24, regulates secondary inflammatory mediators, or affects feedback mechanisms in these cells. Direct analyses of STAT3 and p38 MAPK activation will be necessary to clarify their respective roles.

The inhibitor effects were not uniform. CCL2, CXCL1, and IL-6 were attenuated more consistently, whereas responses involving CXCL3, CXCL10, and IL-8 differed between mRNA and protein analyses and were not uniformly dose-dependent. In particular, the lower inhibitor concentration produced an apparent increase in IL-8 in some comparisons rather than a monotonic suppression. This pattern may reflect compensatory signaling, concentration-dependent off-target effects, or patient variability, but these possibilities cannot be distinguished without direct-pathway and viability measurements. Differences in the significance of IL-24-versus-control protein comparisons between the HJC0152 and SB203580 experiments may similarly reflect their partially overlapping but non-identical specimen sets and biological variability.

This study has several strengths. It used primary fibroblast-enriched synovial cells derived from patients with OA, integrated exploratory transcriptomic screening with targeted validation at both the mRNA and protein levels, and employed paired within-specimen comparisons across treatment conditions. Nevertheless, several limitations should be acknowledged. First, RNA-seq included only two patients, no prospective sample-size calculation was performed, and the Poisson-based within-patients analysis could not estimate population-level biological dispersion; the transcriptomic results are therefore hypothesis-generating. Second, the isolated population was fibroblast-enriched rather than purified, and positive-marker validation was not repeated in the present specimens. Third, the HJC0152 and SB203580 experiments included bilateral specimens from two participants and one participant, respectively, and the cohorts partially overlapped; specimen-level paired analyses did not account for clustering of bilateral knees within participants. Fourth, the present study was not designed to evaluate sex-specific responses to IL-24, and the small number of male patients, together with the use of female specimens only in the inhibitor experiments, precluded meaningful comparisons between women and men. Fifth, no viability assay was performed, so cytotoxicity or generalized metabolic suppression—particularly at 10 μM—cannot be excluded. Sixth, pharmacological inhibitors may have off-target effects, and receptor expression, STAT3 or p38 phosphorylation, genetic perturbation, and temporal pathway activation were not assessed. Finally, the in vitro system does not reproduce the cellular complexity of OA synovium. Despite these limitations, further definition of receptor-proximal signaling and the in vivo relevance of IL-24 may help determine whether IL-24 or selected downstream inflammatory mediators represent tractable targets in molecularly defined OA synovitis.

## 5. Conclusions

In conclusion, IL-24 induced inflammatory chemokine and cytokine responses in the fibroblast-enriched synovial cells derived from patients with knee OA. Exploratory transcriptomic analysis further identified enrichment of inflammatory and immune-related pathways. STAT3 and p38 MAPK inhibition preferentially attenuated IL-24-associated CCL2, CXCL1, and IL-6 responses. These results identify IL-24 as a cytokine capable of inducing inflammatory activation in synovial fibroblasts and provide a basis for further investigation of IL-24-related signaling in OA synovitis.

## Figures and Tables

**Table 1:** Primer sequences used for reverse transcription-quantitative polymerase chain reaction (RT-qPCR).

Gene		Sequence (5′–3′)	bp
*CCL2*	sense	TGCAATCAATGCCCCAGTCA	156
	antisense	GGGTCAGCACAGATCTCCTT	
*CXCL1*	sense	GCTTGCCTCAATCCTGCATC	91
	antisense	AGTTGGATTTGTCACTGTTCAGC	
*CXCL3*	sense	TTGTCTCAACCCCGCATCC	72
	antisense	CAGTTGGTGCTCCCCTTGT	
*CXCL10*	sense	GTGGATGTTCTGACCCTGCT	183
	antisense	GGAGGATGGCAGTGGAAGTC	
*IL6*	sense	GAGGAGACTTGCCTGGTGAAA	199
	antisense	TGGCATTTGTGGTTGGGTCA	
*IL8*	sense	ACACTGCGCCAACACAGAAA	89
	antisense	CAACCCTCTGCACCCAGTTT	
*GAPDH*	sense	TGTTGCCATCAATGACCCCTT	172
	antisense	CTCCACGACGTACTCAGCG	

## Data Availability

The data presented in this study are available on request from the corresponding author.

## Supplementary Materials

The following supporting information can be downloaded at: https://www.mdpi.com/article/10.3390/biomedicines14092084/s1. Figure S1: Time- and dose-dependent induction of CCL2 expression by rhIL-24 in fibroblasts; Table S1: Characteristics of synovial specimens and their allocation to each experimental cohort; Table S2: Overlapping upregulated candidate DEGs in fibroblast-enriched synovial cells from two patients after 6 h of rhIL-24 stimulation; Table S3: Overlapping upregulated candidate DEGs in fibroblast-enriched synovial cells from two patients after 24 h of rhIL-24 stimulation.

## Abbreviations

The following abbreviations are used in this manuscript:

OA	Osteoarthritis
IL	Interleukin
rhIL-24	Recombinant human IL-24
CCL2	C-C motif chemokine ligand 2
CXCL	C-X-C motif chemokine ligand
MMP1	Matrix metallopeptidase 1
TGF-β	Transforming growth factor beta
STAT3	Signal transducer and activator of transcription 3
MAPK	Mitogen-activated protein kinase
RNA-seq	RNA sequencing
cDNA	Complementary DNA
DEGs	Differentially expressed genes
qPCR	Quantitative polymerase chain reaction
ELISA	Enzyme-linked immunosorbent assay
