# Supplementary material for: Interleukin-24 Induces Inflammatory Chemokine and Cytokine Responses in Fibroblast-Enriched Synovial Cells from Patients with Knee Osteoarthritis
Source: Biomedicines. 2026 Sep 16;14(9):2084. doi: 10.3390/biomedicines14092084 (PMC13604332; doi:10.3390/biomedicines14092084)
Supplement: Supplementary file 1 [file biomedicines-14-02084-s001.zip › Figure S1.pdf]

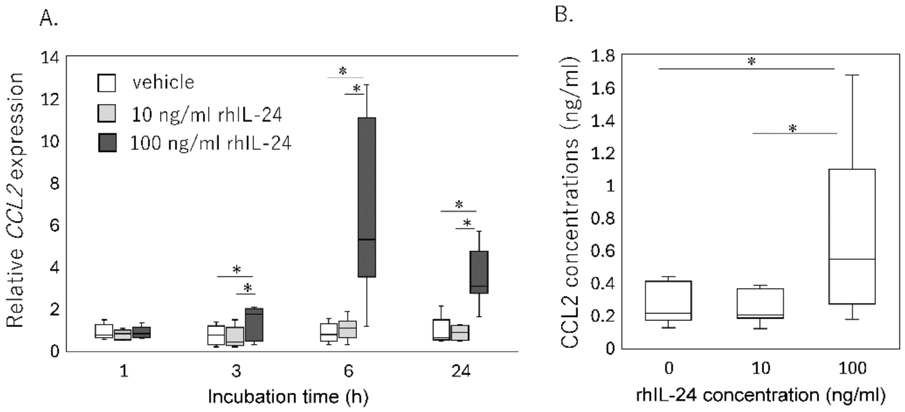

Figure S1. Time- and dose-dependent induction of CCL2 expression by rhIL-24 in fibroblasts. (A) Fibroblasts were treated with vehicle or rhIL-24 (10 or 100 ng/mL) for 1, 3, 6, or 24 h, and relative *CCL2* mRNA expression was evaluated by quantitative PCR. Treatment with 100 ng/mL rhIL-24 significantly increased *CCL2* expression at 3, 6, and 24 h, with a marked induction at 6 h that remained elevated at 24 h.(B) Fibroblasts were treated with rhIL-24 (0, 10, or 100 ng/mL) for 24 h, and CCL2 concentrations in culture supernatants were measured by ELISA. Based on these findings, fibroblasts treated with 100 ng/mL rhIL-24 for 6 and 24 h were used for subsequent RNA-sequencing analysis. Data are presented as box-and-whisker plots. *P < 0.05.
